# Supplementary material for: Bayesian estimation of the measurement of interactions in epidemiological studies
Source: PeerJ. 2024 Mar 29;12:e17128. doi: 10.7717/peerj.17128 (PMC10984183; doi:10.7717/peerj.17128)
Supplement: Supplemental Information 1 [file peerj-12-17128-s001.docx]

**Appendix**

**Appendix A**

**Computation of the additive measure in logistic model based on new reference category**

Considered both factors A and B jointly, then there are four categories: A_0_B_0_ , A_1_B_0_, A_0_B_1_ and A_1_B_1_. To simplify the description, using over-parameterized dummy coding scheme, the coefficients are$\beta_{0}$,$\beta_{1}$, $\beta_{2}$ and $\beta_{3}$ in logistic regression respectively. When using A_0_B_0_ as the reference category ($\beta_{0}=0$ in dummy coding scheme), The ORs can be rewritten as $OR_{00}=e^{\beta_{0}-\beta_{0}}$, $OR_{10}=e^{\beta_{1}-\beta_{0}}$, $OR_{01}=e^{\beta_{2}-\beta_{0}}$ and $OR_{11}=e^{\beta_{3}-\beta_{0}}$ respectively. The three measures of interaction can be equivalently written as follows

$RERI=OR_{11}-OR_{10}-OR_{01}+OR_{00}=e^{-\beta_{0}}\cdot(e^{\beta_{3}}-e^{\beta_{1}}-e^{\beta_{2}}+e^{\beta_{0}})$,

$AP=e^{-\beta_{0}}\cdot(e^{\beta_{3}}-e^{\beta_{1}}-e^{\beta_{2}}+e^{\beta_{0}})/e^{\beta_{3}-\beta_{0}}=e^{-\beta_{3}}\cdot(e^{\beta_{3}}-e^{\beta_{1}}-e^{\beta_{2}}+e^{\beta_{0}})$,

$S=\frac{e^{\beta_{3}}-e^{\beta_{0}}}{e^{\beta_{1}}+e^{\beta_{2}}-2e^{\beta_{0}}}$.

when choosing the category with the lowest risk when both factors are considered jointly as the reference category, for example the group A_1_B_0_ , the coefficients $\beta'$s of the four categories can be expressed as $\beta'_{0}=\beta_{0}-\beta_{1}$, $\beta'_{1}=\beta_{1}-\beta_{1}$, $\beta'_{2}=\beta_{2}-\beta_{1}$ and $\beta'_{3}=\beta_{3}-\beta_{1}$. Then the ORs can be expressed as $OR'_{00}=e^{\beta_{0}-\beta_{1}}$, $OR'_{10}=e^{\beta_{1}-\beta_{1}}$, $OR'_{01}=e^{\beta_{2}-\beta_{1}}$ and $OR'_{11}=e^{\beta_{3}-\beta_{1}}$ respectively. The three measures of interaction can be computed by rearranging the ORs, which have the form of

$RERI=OR'_{01}-OR'_{00}-OR'_{11}+OR'_{10}=-e^{-\beta_{1}}\cdot(e^{\beta_{3}}-e^{\beta_{1}}-e^{\beta_{2}}+e^{\beta_{0}})$,

$AP=RERI/OR'_{01}=-e^{-\beta_{2}}\cdot(e^{\beta_{3}}-e^{\beta_{1}}-e^{\beta_{2}}+e^{\beta_{0}})$,

$S=\frac{e^{\beta_{2}}-e^{\beta_{1}}}{e^{\beta_{3}}+e^{\beta_{0}}-2e^{\beta_{1}}}$.

So does the reference category set as group A_0_B_1_or A_1_B_1_. When the reference category is A_0_B_1_, the three measures of interaction have the form of

$RERI=OR'_{10}-OR'_{11}-OR'_{00}+OR'_{01}=-e^{-\beta_{2}}\cdot(e^{\beta_{3}}-e^{\beta_{1}}-e^{\beta_{2}}+e^{\beta_{0}})$,

$AP=RERI/OR'_{10}=-e^{-\beta_{1}}\cdot(e^{\beta_{3}}-e^{\beta_{1}}-e^{\beta_{2}}+e^{\beta_{0}})$,

$S=\frac{e^{\beta_{1}}-e^{\beta_{2}}}{e^{\beta_{3}}+e^{\beta_{0}}-2e^{\beta_{2}}}$.

When the reference category is A_1_B_1_, the three measures of interaction have the form of

$RERI=OR'_{00}-OR'_{01}-OR'_{10}+OR'_{11}=e^{-\beta_{3}}\cdot(e^{\beta_{3}}-e^{\beta_{1}}-e^{\beta_{2}}+e^{\beta_{0}})$,

$AP=RERI/OR'_{00}=e^{-\beta_{0}}\cdot(e^{\beta_{3}}-e^{\beta_{1}}-e^{\beta_{2}}+e^{\beta_{0}})$,

$S=\frac{e^{\beta_{0}}-e^{\beta_{3}}}{e^{\beta_{1}}+e^{\beta_{2}}-2e^{\beta_{3}}}$.

**Appendix B**

***RR* can be expressed as a function of both *OR* and the intercept *b*_0_.**

If there are independent variable *X* and dependent variable *Y* in logistic regression, then we have:

$\log it\text{ }P(Y=1)=b_{0}+b_{1}X$;

The probability of an event occurring in the control group (*X*=0) is:

$P_{0}=P(Y=1|X=0)=\frac{e^{b_{0}}}{1+e^{b_{0}}}$;

The probability of an event occurring in the treatment group (*X*=1) is:

$P_{1}=P(Y=1|X=1)=\frac{e^{b_{0}+b_{1}}}{1+e^{b_{0}+b_{1}}}$;

The *OR* value can be expressed as:

$OR=\frac{P_{1}/(1-P_{1})}{P_{0}/(1-P_{0})}=\frac{e^{b_{0}+b_{1}}}{e^{b_{0}}}=e^{b_{1}}$;

The *RR* value can be expressed as:

$RR=\frac{P_{1}}{P_{0}}=\frac{e^{b_{1}}(1+e^{b_{0}})}{1+e^{b_{0}+b_{1}}}=\frac{OR\cdot(1+e^{b_{0}})}{1+OR\cdot e^{b_{0}}}=\frac{OR}{1-P_{0}+P_{0}\cdot OR}$.

**Appendix C**

**R function for computing new coefficients based on the lowest risk category as reference category**

newCoefficents <- function(coefficients){ # coefficients: the original coefficients

if(length(coefficients)!=4) stop("the length of coefficients must be equate to 4")

minloc <- which.min(coefficients)

loc <- 1:4

if(minloc==2) loc<-c(2,1,4,3)

if(minloc==3) loc<-c(3,4,1,2)

if(minloc==4) loc<-c(4,3,2,1)

new.coef<- coefficients[loc]

new.coef<- new.coef – new.coef[1]

return(new.coef)

}
