# Supplementary material for: Bayesian estimation of the measurement of interactions in epidemiological studies
Source: PeerJ. 2024 Mar 29;12:e17128. doi: 10.7717/peerj.17128 (PMC10984183; doi:10.7717/peerj.17128)
Supplement: Supplemental Information 2 [file peerj-12-17128-s002.docx]

Table S1. Coverage properties of the 95% two-sided confidence intervals for RERI in unbalanced design study

|  | delta | | | |  | bootstrap | | | |  | bayesian | | | |
| --- | --- | --- | --- | --- | --- | --- | --- | --- | --- | --- | --- | --- | --- | --- |
| RERI | Estimate | Left | Cover | Right |  | Estimate | Left | Cover | Right |  | Estimate | Left | Cover | Right |
| 12.00 | 11.56 | 7.2 | 92.8 | 0.0 |  | 11.71 | 2.2 | 94.4 | 3.4 |  | 12.01 | 2.4 | 94.6 | 3.0 |
| 4.00 | 4.03 | 4.7 | 95.3 | 0.0 |  | 3.98 | 2.4 | 94.7 | 2.9 |  | 4.17 | 2.5 | 94.7 | 2.8 |
| 0.00 | 0.00 | 2.6 | 97.4 | 0.0 |  | -0.10 | 3.0 | 94.5 | 2.5 |  | -0.04 | 2.9 | 94.6 | 2.5 |
| -2.00 | -1.97 | 2.1 | 97.4 | 0.5 |  | -2.02 | 3.4 | 94.0 | 2.6 |  | -1.98 | 3.6 | 93.8 | 2.6 |
| -4.00 | -4.12 | 0.8 | 96.9 | 2.3 |  | -4.18 | 4.0 | 94.3 | 1.7 |  | -4.15 | 4.1 | 94.3 | 1.6 |
|  |  |  |  |  |  |  |  |  |  |  |  |  |  |  |
| 9.00 | 8.76 | 9.3 | 90.7 | 0.0 |  | 8.86 | 3.7 | 93.4 | 2.9 |  | 9.10 | 4.1 | 93.4 | 2.5 |
| 3.00 | 2.92 | 6.1 | 93.9 | 0.0 |  | 2.90 | 4.3 | 93.2 | 2.5 |  | 3.00 | 4.5 | 93.4 | 2.1 |
| 0.00 | -0.05 | 2.0 | 97.8 | 0.2 |  | -0.10 | 2.2 | 95.5 | 2.3 |  | -0.11 | 2.6 | 95.3 | 2.1 |
| -1.50 | -1.55 | 1.3 | 97.5 | 1.2 |  | -1.57 | 2.2 | 95.5 | 2.3 |  | -1.59 | 2.2 | 95.6 | 2.2 |
| -3.00 | -3.09 | 1.0 | 96.4 | 2.6 |  | -3.11 | 3.0 | 94.6 | 2.4 |  | -3.14 | 2.8 | 95.1 | 2.1 |
|  |  |  |  |  |  |  |  |  |  |  |  |  |  |  |
| 8.25 | 8.34 | 6.5 | 93.5 | 0.0 |  | 8.38 | 2.3 | 94.3 | 3.4 |  | 8.60 | 2.8 | 94.5 | 2.7 |
| 2.75 | 2.80 | 3.7 | 96.3 | 0.0 |  | 2.79 | 2.6 | 94.8 | 2.6 |  | 2.84 | 2.9 | 94.2 | 2.9 |
| 0.00 | 0.00 | 1.9 | 97.9 | 0.2 |  | -0.03 | 2.7 | 94.3 | 3.0 |  | 0.01 | 2.8 | 94.0 | 3.2 |
| -1.38 | -1.38 | 0.5 | 97.2 | 2.3 |  | -1.40 | 1.9 | 94.7 | 3.4 |  | -1.39 | 2.0 | 94.5 | 3.5 |
| -2.75 | -2.77 | 0.4 | 97.3 | 2.3 |  | -2.81 | 2.6 | 95.6 | 1.8 |  | -2.81 | 2.2 | 95.8 | 2.0 |
|  |  |  |  |  |  |  |  |  |  |  |  |  |  |  |
| 5.25 | 5.20 | 5.9 | 94.1 | 0.0 |  | 5.23 | 3.3 | 93.3 | 3.4 |  | 5.33 | 3.5 | 93.3 | 3.2 |
| 1.75 | 1.77 | 4.1 | 95.9 | 0.0 |  | 1.73 | 3.1 | 93.9 | 3.0 |  | 1.76 | 3.3 | 94.1 | 2.6 |
| 0.00 | 0.01 | 1.8 | 97.8 | 0.4 |  | -0.02 | 2.2 | 95.4 | 2.4 |  | -0.03 | 2.3 | 95.3 | 2.4 |
| -0.88 | -0.92 | 1.1 | 97.5 | 1.4 |  | -0.93 | 2.4 | 95.2 | 2.4 |  | -0.95 | 2.4 | 95.7 | 1.9 |
| -1.75 | -1.75 | 0.5 | 95.4 | 4.1 |  | -1.80 | 2.4 | 95.2 | 2.4 |  | -1.80 | 3.0 | 94.6 | 2.4 |

Table S2. Coverage properties of the 95% two-sided confidence intervals for AP in unbalanced design study

|  | delta | | | |  | bootstrap | | | |  | bayesian | | | |
| --- | --- | --- | --- | --- | --- | --- | --- | --- | --- | --- | --- | --- | --- | --- |
| AP | Estimate | Left | Cover | Right |  | Estimate | Left | Cover | Right |  | Estimate | Left | Cover | Right |
| 0.60 | 0.59 | 0.2 | 92.6 | 7.2 |  | 0.59 | 2.0 | 95.6 | 2.4 |  | 0.60 | 2.5 | 95.3 | 2.2 |
| 0.33 | 0.32 | 0.1 | 93.3 | 6.6 |  | 0.32 | 2.3 | 95 | 2.7 |  | 0.33 | 2.7 | 94.8 | 2.5 |
| 0.00 | 0.00 | 0.3 | 94.2 | 5.5 |  | -0.01 | 3.0 | 94.5 | 2.5 |  | 0.00 | 2.9 | 94.6 | 2.5 |
| -0.33 | -0.33 | 0.3 | 92.9 | 6.8 |  | -0.34 | 2.7 | 94.8 | 2.5 |  | -0.33 | 3.3 | 94.2 | 2.5 |
| -1.00 | -1.03 | 0.2 | 92.9 | 6.9 |  | -1.05 | 3.3 | 94.8 | 1.9 |  | -1.05 | 2.9 | 95.6 | 1.5 |
|  |  |  |  |  |  |  |  |  |  |  |  |  |  |  |
| 0.60 | 0.60 | 2.0 | 91.8 | 6.2 |  | 0.60 | 4.2 | 93.6 | 2.2 |  | 0.60 | 4.9 | 93.3 | 1.8 |
| 0.33 | 0.32 | 1.3 | 92.0 | 6.7 |  | 0.32 | 4.3 | 93.6 | 2.1 |  | 0.33 | 5.0 | 93.4 | 1.6 |
| 0.00 | -0.01 | 0.2 | 93.0 | 6.8 |  | -0.01 | 2.2 | 95.5 | 2.3 |  | -0.02 | 2.6 | 95.3 | 2.1 |
| -0.33 | -0.35 | 0.1 | 92.8 | 7.1 |  | -0.36 | 2.1 | 95.1 | 2.8 |  | -0.36 | 2.4 | 95.3 | 2.3 |
| -1.00 | -1.02 | 0.3 | 91.9 | 7.8 |  | -1.05 | 3.5 | 93.9 | 2.6 |  | -1.06 | 3.8 | 94.1 | 2.1 |
|  |  |  |  |  |  |  |  |  |  |  |  |  |  |  |
| 0.60 | 0.60 | 0.1 | 91.2 | 8.7 |  | 0.60 | 2.5 | 94.6 | 2.9 |  | 0.61 | 3.1 | 93.7 | 3.2 |
| 0.33 | 0.34 | 0.2 | 91.2 | 8.6 |  | 0.33 | 2.4 | 94.4 | 3.2 |  | 0.34 | 2.7 | 94.1 | 3.2 |
| 0.00 | 0.00 | 0.1 | 90.5 | 9.4 |  | -0.01 | 2.7 | 94.3 | 3.0 |  | 0.00 | 2.8 | 94.0 | 3.2 |
| -0.33 | -0.35 | 0.2 | 91.3 | 8.5 |  | -0.36 | 2.3 | 95.0 | 2.7 |  | -0.36 | 2.7 | 94.6 | 2.7 |
| -1.00 | -1.00 | 0.4 | 93.3 | 6.3 |  | -1.03 | 2.5 | 96.0 | 1.5 |  | -1.02 | 2.3 | 96.2 | 1.5 |
|  |  |  |  |  |  |  |  |  |  |  |  |  |  |  |
| 0.60 | 0.60 | 1.4 | 89.2 | 9.4 |  | 0.61 | 2.3 | 94.5 | 3.2 |  | 0.61 | 2.9 | 94.5 | 2.6 |
| 0.33 | 0.34 | 1.1 | 90.3 | 8.6 |  | 0.34 | 2.8 | 94.6 | 2.6 |  | 0.33 | 3.0 | 94.8 | 2.2 |
| 0.00 | 0.00 | 0.1 | 91.4 | 8.5 |  | -0.01 | 2.2 | 95.4 | 2.4 |  | -0.01 | 2.3 | 95.3 | 2.4 |
| -0.33 | -0.36 | 0.1 | 91.3 | 8.6 |  | -0.36 | 2.4 | 95.4 | 2.2 |  | -0.37 | 2.2 | 95.5 | 2.3 |
| -1.00 | -1.04 | 1.9 | 88.4 | 9.7 |  | -1.05 | 3.1 | 94.3 | 2.6 |  | -1.03 | 3.6 | 94.2 | 2.2 |

Table S3. Coverage properties of the 95% two-sided confidence intervals for S in unbalanced design study

|  | delta | | | |  | bootstrap | | | |  | bayesian | | | |
| --- | --- | --- | --- | --- | --- | --- | --- | --- | --- | --- | --- | --- | --- | --- |
| S | Estimate | Left | Cover | Right |  | Estimate | Left | Cover | Right |  | Estimate | Left | Cover | Right |
| 2.71 | 2.64 | 2.7 | 96.0 | 1.3 |  | 2.64 | 1.9 | 95.5 | 2.6 |  | 2.69 | 2.1 | 95.7 | 2.2 |
| 1.57 | 1.54 | 2.5 | 96.0 | 1.5 |  | 1.54 | 2.4 | 94.9 | 2.7 |  | 1.56 | 2.6 | 94.9 | 2.5 |
| 1.00 | 1.00 | 2.5 | 95.5 | 2.0 |  | 0.99 | 3.0 | 94.5 | 2.5 |  | 1.00 | 2.9 | 94.6 | 2.5 |
| 0.71 | 0.72 | 2.1 | 95.4 | 2.5 |  | 0.71 | 2.7 | 94.7 | 2.6 |  | 0.71 | 3.0 | 94.3 | 2.7 |
| 0.43 | 0.42 | 1.6 | 96.0 | 2.4 |  | 0.41 | 3.1 | 94.9 | 2.0 |  | 0.41 | 3.3 | 95.1 | 1.6 |
|  |  |  |  |  |  |  |  |  |  |  |  |  |  |  |
| 2.80 | 2.75 | 5.0 | 94.2 | 0.8 |  | 2.79 | 4.2 | 93.6 | 2.2 |  | 2.80 | 5.0 | 93.3 | 1.7 |
| 1.60 | 1.58 | 4.3 | 94.4 | 1.3 |  | 1.58 | 4.3 | 93.6 | 2.1 |  | 1.58 | 4.6 | 93.7 | 1.7 |
| 1.00 | 0.99 | 2.2 | 96.0 | 1.8 |  | 0.98 | 2.2 | 95.6 | 2.2 |  | 0.98 | 2.6 | 95.3 | 2.1 |
| 0.70 | 0.69 | 1.6 | 96.2 | 2.2 |  | 0.68 | 2.0 | 95.5 | 2.5 |  | 0.68 | 2.2 | 95.4 | 2.4 |
| 0.40 | 0.40 | 0.2 | 97.3 | 2.5 |  | 0.39 | 3.6 | 94.6 | 1.8 |  | 0.39 | 3.5 | 94.7 | 1.8 |
|  |  |  |  |  |  |  |  |  |  |  |  |  |  |  |
| 2.83 | 2.81 | 3.0 | 95.4 | 1.6 |  | 2.85 | 2.3 | 94.5 | 3.2 |  | 2.86 | 2.7 | 94.0 | 3.3 |
| 1.61 | 1.62 | 2.3 | 95.3 | 2.4 |  | 1.61 | 2.3 | 94.5 | 3.2 |  | 1.63 | 2.4 | 94.3 | 3.3 |
| 1.00 | 1.00 | 2.3 | 94.9 | 2.8 |  | 0.99 | 2.7 | 94.3 | 3.0 |  | 1.00 | 2.8 | 94.0 | 3.2 |
| 0.69 | 0.68 | 1.0 | 96.2 | 2.8 |  | 0.68 | 2.4 | 94.9 | 2.7 |  | 0.68 | 2.6 | 94.6 | 2.8 |
| 0.39 | 0.38 | 0.4 | 98.0 | 1.6 |  | 0.37 | 2.8 | 95.6 | 1.6 |  | 0.37 | 2.8 | 95.7 | 1.5 |
|  |  |  |  |  |  |  |  |  |  |  |  |  |  |  |
| 3.10 | 3.11 | 2.5 | 96.3 | 1.2 |  | 3.16 | 2.2 | 95.5 | 2.3 |  | 3.14 | 2.5 | 95.1 | 2.4 |
| 1.70 | 1.73 | 2.9 | 95.6 | 1.5 |  | 1.73 | 2.7 | 95.3 | 2.0 |  | 1.71 | 3.1 | 94.7 | 2.2 |
| 1.00 | 1.01 | 1.2 | 97.0 | 1.8 |  | 0.99 | 2.2 | 96.1 | 1.7 |  | 0.99 | 2.3 | 95.3 | 2.4 |
| 0.65 | 0.63 | 0.2 | 97.4 | 2.4 |  | 0.63 | 2.5 | 95.8 | 1.7 |  | 0.62 | 2.3 | 95.3 | 2.4 |
| 0.30 | 0.29 | 0.1 | 97.2 | 2.7 |  | 0.28 | 3.0 | 94.9 | 2.1 |  | 0.29 | 3.1 | 94.0 | 2.9 |

Table S4. Coverage properties of the 95% two-sided confidence intervals for RERI in balanced design with a confounding variable study

|  | delta | | | |  | bootstrap | | | |  | bayesian | | | |
| --- | --- | --- | --- | --- | --- | --- | --- | --- | --- | --- | --- | --- | --- | --- |
| RERI | Estimate | Left | Cover | Right |  | Estimate | Left | Cover | Right |  | Estimate | Left | Cover | Right |
| 12.00 | 11.97 | 6.0 | 94.0 | 0.0 |  | 12.28 | 1.5 | 93.9 | 4.6 |  | 12.93 | 2.1 | 94.0 | 3.9 |
| 4.00 | 3.93 | 5.0 | 95.0 | 0.0 |  | 3.85 | 1.8 | 94.9 | 3.3 |  | 4.10 | 3.0 | 94.5 | 2.5 |
| 0.00 | -0.21 | 2.5 | 97.5 | 0.0 |  | -0.26 | 2.5 | 94.8 | 2.7 |  | -0.20 | 3.3 | 94.3 | 2.4 |
| -2.00 | -2.03 | 1.4 | 98.5 | 0.1 |  | -2.08 | 2.7 | 94.7 | 2.6 |  | -2.06 | 3.4 | 94.4 | 2.2 |
| -4.00 | -4.12 | 0.8 | 97.2 | 2.0 |  | -4.24 | 2.8 | 95.3 | 1.9 |  | -4.21 | 3.4 | 94.4 | 2.2 |
|  |  |  |  |  |  |  |  |  |  |  |  |  |  |  |
| 9.00 | 9.06 | 6.9 | 93.1 | 0.0 |  | 9.22 | 2.6 | 92.9 | 4.5 |  | 9.55 | 3.2 | 92.6 | 4.2 |
| 3.00 | 2.95 | 4.4 | 95.6 | 0.0 |  | 3.00 | 2.5 | 94.2 | 3.3 |  | 3.11 | 3.3 | 93.6 | 3.1 |
| 0.00 | 0.00 | 2.3 | 97.7 | 0.0 |  | -0.02 | 2.5 | 95.1 | 2.4 |  | 0.02 | 2.9 | 94.8 | 2.3 |
| -1.50 | -1.55 | 1.6 | 97.9 | 0.5 |  | -1.60 | 2.8 | 94.6 | 2.6 |  | -1.59 | 3.4 | 94.0 | 2.6 |
| -3.00 | -2.97 | 0.8 | 96.4 | 2.8 |  | -3.00 | 3.1 | 94.1 | 2.8 |  | -3.01 | 3.6 | 93.6 | 2.8 |
|  |  |  |  |  |  |  |  |  |  |  |  |  |  |  |
| 8.25 | 8.18 | 6.1 | 93.9 | 0.0 |  | 8.25 | 1.4 | 94.4 | 4.2 |  | 8.67 | 1.8 | 94.9 | 3.3 |
| 2.75 | 2.51 | 3.6 | 96.4 | 0.0 |  | 2.49 | 1.9 | 95.1 | 3.0 |  | 2.55 | 2.7 | 94.6 | 2.7 |
| 0.00 | -0.14 | 1.0 | 99.0 | 0.0 |  | -0.18 | 2.1 | 95.3 | 2.6 |  | -0.13 | 2.7 | 95.0 | 2.3 |
| -1.38 | -1.57 | 0.7 | 99.2 | 0.1 |  | -1.62 | 3.0 | 94.1 | 2.9 |  | -1.59 | 3.6 | 94.0 | 2.4 |
| -2.75 | -2.84 | 0.2 | 97.1 | 2.7 |  | -2.90 | 4.1 | 93.7 | 2.2 |  | -2.88 | 4.7 | 93.3 | 2.0 |
|  |  |  |  |  |  |  |  |  |  |  |  |  |  |  |
| 5.25 | 5.02 | 6.8 | 93.2 | 0.0 |  | 5.09 | 2.8 | 93.3 | 3.9 |  | 5.17 | 3.3 | 93.6 | 3.1 |
| 1.75 | 1.55 | 4.4 | 95.6 | 0.0 |  | 1.53 | 2.5 | 95.0 | 2.5 |  | 1.56 | 3.0 | 94.3 | 2.7 |
| 0.00 | -0.11 | 1.7 | 98.1 | 0.2 |  | -0.14 | 2.1 | 95.4 | 2.5 |  | -0.14 | 2.8 | 94.9 | 2.3 |
| -0.88 | -0.87 | 1.0 | 98.2 | 0.8 |  | -0.91 | 2.7 | 95.4 | 1.9 |  | -0.92 | 3.5 | 94.7 | 1.8 |
| -1.75 | -1.80 | 0.7 | 96.4 | 2.9 |  | -1.84 | 2.4 | 95.3 | 2.3 |  | -1.84 | 3.3 | 94.8 | 1.9 |

Table S5. Coverage properties of the 95% two-sided confidence intervals for AP in balanced design with a confounding variable study

|  | delta | | | |  | bootstrap | | | |  | bayesian | | | |
| --- | --- | --- | --- | --- | --- | --- | --- | --- | --- | --- | --- | --- | --- | --- |
| AP | Estimate | Left | Cover | Right |  | Estimate | Left | Cover | Right |  | Estimate | Left | Cover | Right |
| 0.60 | 0.59 | 0.0 | 91.2 | 8.8 |  | 0.60 | 1.5 | 94.4 | 4.1 |  | 0.61 | 2.4 | 94.3 | 3.3 |
| 0.33 | 0.33 | 0.1 | 91.7 | 8.2 |  | 0.33 | 2.1 | 95.4 | 2.5 |  | 0.34 | 3.1 | 94.4 | 2.5 |
| 0.00 | -0.03 | 0.2 | 91.7 | 8.1 |  | -0.04 | 2.5 | 94.8 | 2.7 |  | -0.02 | 3.3 | 94.3 | 2.4 |
| -0.33 | -0.34 | 0.2 | 92.4 | 7.4 |  | -0.35 | 2.5 | 94.8 | 2.7 |  | -0.34 | 3.0 | 94.2 | 2.8 |
| -1.00 | -1.03 | 0.3 | 91.1 | 8.6 |  | -1.06 | 2.6 | 94.5 | 2.9 |  | -1.05 | 3.6 | 93.7 | 2.7 |
|  |  |  |  |  |  |  |  |  |  |  |  |  |  |  |
| 0.60 | 0.60 | 0.5 | 91.6 | 7.9 |  | 0.61 | 3.0 | 93.3 | 3.7 |  | 0.61 | 3.9 | 92.8 | 3.3 |
| 0.33 | 0.34 | 0.3 | 92.6 | 7.1 |  | 0.34 | 2.5 | 94.8 | 2.7 |  | 0.34 | 3.3 | 94.3 | 2.4 |
| 0.00 | 0.00 | 0.2 | 90.8 | 9.0 |  | 0.00 | 2.5 | 95.1 | 2.4 |  | 0.00 | 2.9 | 94.8 | 2.3 |
| -0.33 | -0.36 | 0.2 | 92.2 | 7.6 |  | -0.38 | 2.8 | 94.6 | 2.6 |  | -0.37 | 3.6 | 93.8 | 2.6 |
| -1.00 | -1.00 | 0.1 | 91.6 | 8.3 |  | -1.04 | 3.7 | 92.8 | 3.5 |  | -1.03 | 3.7 | 92.9 | 3.4 |
|  |  |  |  |  |  |  |  |  |  |  |  |  |  |  |
| 0.60 | 0.60 | 0.0 | 90.9 | 9.1 |  | 0.60 | 1.2 | 94.3 | 4.5 |  | 0.61 | 1.8 | 94.6 | 3.6 |
| 0.33 | 0.31 | 0.1 | 91.4 | 8.5 |  | 0.31 | 1.6 | 95.4 | 3.0 |  | 0.32 | 2.7 | 94.6 | 2.7 |
| 0.00 | -0.03 | 0.1 | 90.8 | 9.1 |  | -0.03 | 2.1 | 95.3 | 2.6 |  | -0.02 | 2.7 | 95.0 | 2.3 |
| -0.33 | -0.39 | 0.2 | 90.3 | 9.5 |  | -0.40 | 3.0 | 93.9 | 3.1 |  | -0.39 | 3.4 | 93.9 | 2.7 |
| -1.00 | -1.03 | 0.1 | 91.2 | 8.7 |  | -1.08 | 2.9 | 94.8 | 2.3 |  | -1.06 | 3.8 | 94.1 | 2.1 |
|  |  |  |  |  |  |  |  |  |  |  |  |  |  |  |
| 0.60 | 0.59 | 1.1 | 90.6 | 8.3 |  | 0.59 | 2.5 | 94.6 | 2.9 |  | 0.60 | 3.7 | 93.6 | 2.7 |
| 0.33 | 0.31 | 1.0 | 91.0 | 8.0 |  | 0.31 | 2.5 | 95.3 | 2.2 |  | 0.31 | 3.4 | 94.3 | 2.3 |
| 0.00 | -0.04 | 0.2 | 90.7 | 9.1 |  | -0.05 | 2.1 | 95.4 | 2.5 |  | -0.04 | 2.8 | 94.9 | 2.3 |
| -0.33 | -0.34 | 0.0 | 91.5 | 8.5 |  | -0.37 | 2.6 | 95.5 | 1.9 |  | -0.36 | 3.4 | 95.1 | 1.5 |
| -1.00 | -1.02 | 2.8 | 87.6 | 9.6 |  | -1.06 | 4.2 | 93.4 | 2.4 |  | -1.03 | 4.3 | 93.6 | 2.1 |

Table S6. Coverage properties of the 95% two-sided confidence intervals for S in balanced design with a confounding variable study

|  | delta | | | |  | bootstrap | | | |  | bayesian | | | |
| --- | --- | --- | --- | --- | --- | --- | --- | --- | --- | --- | --- | --- | --- | --- |
| S | Estimate | Left | Cover | Right |  | Estimate | Left | Cover | Right |  | Estimate | Left | Cover | Right |
| 2.71 | 2.69 | 2.7 | 96.4 | 0.9 |  | 2.69 | 1.7 | 94.5 | 3.8 |  | 2.79 | 2.5 | 94.1 | 3.4 |
| 1.57 | 1.55 | 2.9 | 95.5 | 1.6 |  | 1.56 | 2.1 | 95.4 | 2.5 |  | 1.57 | 3.1 | 94.5 | 2.4 |
| 1.00 | 0.97 | 2.5 | 95.5 | 2.0 |  | 0.96 | 2.5 | 94.8 | 2.7 |  | 0.97 | 3.3 | 94.3 | 2.4 |
| 0.71 | 0.71 | 1.8 | 95.8 | 2.4 |  | 0.70 | 2.7 | 94.3 | 3.0 |  | 0.71 | 3.2 | 94.0 | 2.8 |
| 0.43 | 0.42 | 1.2 | 95.7 | 3.1 |  | 0.41 | 2.3 | 94.8 | 2.9 |  | 0.42 | 3.2 | 94.3 | 2.5 |
|  |  |  |  |  |  |  |  |  |  |  |  |  |  |  |
| 2.80 | 2.80 | 3.7 | 94.8 | 1.5 |  | 2.87 | 2.8 | 93.4 | 3.8 |  | 2.89 | 3.5 | 93.2 | 3.3 |
| 1.60 | 1.61 | 3.0 | 95.2 | 1.8 |  | 1.62 | 2.4 | 94.8 | 2.8 |  | 1.62 | 3.1 | 94.4 | 2.5 |
| 1.00 | 1.00 | 2.4 | 96.0 | 1.6 |  | 1.00 | 2.5 | 95.1 | 2.4 |  | 1.00 | 2.9 | 94.8 | 2.3 |
| 0.70 | 0.68 | 2.0 | 95.5 | 2.5 |  | 0.67 | 3.0 | 94.2 | 2.8 |  | 0.67 | 4.0 | 93.5 | 2.5 |
| 0.40 | 0.40 | 1.0 | 95.0 | 4.0 |  | 0.39 | 3.6 | 93.1 | 3.3 |  | 0.39 | 4.0 | 92.3 | 3.7 |
|  |  |  |  |  |  |  |  |  |  |  |  |  |  |  |
| 2.83 | 2.84 | 2.3 | 96.0 | 1.7 |  | 2.87 | 1.4 | 94.3 | 4.3 |  | 2.93 | 1.9 | 94.6 | 3.5 |
| 1.61 | 1.56 | 2.3 | 95.6 | 2.1 |  | 1.56 | 1.9 | 95.1 | 3.0 |  | 1.58 | 2.6 | 94.9 | 2.5 |
| 1.00 | 0.97 | 1.9 | 95.9 | 2.2 |  | 0.96 | 2.1 | 95.3 | 2.6 |  | 0.97 | 2.7 | 95.0 | 2.3 |
| 0.69 | 0.66 | 1.7 | 95.6 | 2.7 |  | 0.65 | 2.9 | 94.4 | 2.7 |  | 0.66 | 3.4 | 93.9 | 2.7 |
| 0.39 | 0.37 | 0.7 | 95.9 | 3.4 |  | 0.36 | 3.0 | 94.5 | 2.5 |  | 0.36 | 3.5 | 93.9 | 2.6 |
|  |  |  |  |  |  |  |  |  |  |  |  |  |  |  |
| 3.10 | 3.00 | 3.2 | 95.4 | 1.4 |  | 3.08 | 2.3 | 95.4 | 2.3 |  | 3.07 | 3.4 | 94.1 | 2.5 |
| 1.70 | 1.63 | 2.7 | 95.7 | 1.6 |  | 1.64 | 2.4 | 95.9 | 1.7 |  | 1.64 | 3.2 | 94.7 | 2.1 |
| 1.00 | 0.95 | 1.7 | 96.3 | 2.0 |  | 0.94 | 2.1 | 96.0 | 1.9 |  | 0.95 | 2.8 | 94.9 | 2.3 |
| 0.65 | 0.63 | 0.2 | 98.3 | 1.5 |  | 0.62 | 2.5 | 95.8 | 1.7 |  | 0.63 | 3.2 | 95.1 | 1.7 |
| 0.30 | 0.30 | 0.0 | 97.0 | 3.0 |  | 0.28 | 5.1 | 93.7 | 1.2 |  | 0.29 | 4.3 | 93.5 | 2.2 |
